# Genetic diversity of Nile tilapia (*Oreochromis niloticus*) populations in Ethiopia: insights from nuclear DNA microsatellites and implications for conservation

**DOI:** 10.1186/s12862-021-01829-2

**Published:** 2021-06-07

**Authors:** Genanaw Tesfaye, Manuel Curto, Paul Meulenbroek, Gernot K. Englmaier, Papius Dias Tibihika, Esayas Alemayehu, Abebe Getahun, Harald Meimberg

**Affiliations:** 1 EIAR - National Fisheries and Other Aquatic Life Research Center, P.O. Box 64, Sebeta, Ethiopia; 2 Institute for Integrative Nature Conservation Research, University of Natural Resources and Life Sciences, Vienna, Gregor Mendel-Straße 33, 1180 Vienna, Austria; 3 MARE-Marine and Environmental Sciences Centre, Universidade de Lisboa, Lisbon, Portugal; 4 Institute of Hydrobiology and Aquatic Ecosystem Management (IHG), University of Natural Resources and Life Sciences, Vienna, Gregor-Mendel Straße 33, 1180 Vienna, Austria; 5 WasserCluster Lunz – biologische Station, Lunz am See, Dr. Carl Kupelwieser Prom. 5, 3293 Lunz/See, Austria; 6 University of Graz, Institute of Biology, Universitätsplatz 2, 8010 Graz, Austria; 7 National Environment Management Authority, P.O. Box 22255, Kampala, Uganda; 8 Department of Zoological Sciences, Addis Ababa University, 1000 Addis Ababa, Ethiopia

**Keywords:** Ethiopian Rift Valley, Cichlid, Genotyping, Admixture, Freshwater biodiversity, Stocking

## Abstract

**Background:**

Nile tilapia, *Oreochromis niloticus* (Linnaeus, 1758) is among the economically most important freshwater fish species in East Africa, and a major source of protein for local consumption. Human induced translocations of non-native stocks for aquaculture and fisheries have been found as a potential threat to the genetic diversity and integrity of local populations. In the present study, we investigate the genetic structure of *O. niloticus* from 16 waterbodies across Ethiopia using 37 microsatellite loci with SSR-GBAS techniques.

**Results:**

The samples are structured into three main clusters shaped either by biogeographic factors or stocking activities. High *F*_*ST*_ values (Global *F*_*ST*_ = 0.438) between populations indicate a high level of genetic differentiation and may suggest long term isolation even within the same drainage systems. Natural populations of the Omo-Turkana system and the lakes in the Southern Main Ethiopian Rift showed the highest genetic variability while low variability was found in stocked populations of lakes Hora, Hashenge and Hayq.

**Conclusions:**

The results presented herein, may provide an essential basis for the management and conservation of the unique genetic resources in northern East Africa, and advance our understanding of biodiversity, phylogeny, evolution and development towards phylogenetically more accurate taxonomic classifications.

**Supplementary Information:**

The online version contains supplementary material available at 10.1186/s12862-021-01829-2.

## Background

Nile tilapia, *Oreochromis niloticus* (Linnaeus, 1758), is native to East, Central and West Africa, as well as to the Middle East, particularly in the Jordan valley [62, 68]. In East Africa, the species is found in water bodies of both the Eastern and Western Rift Valley [2, 52]. Due to its high importance for aquaculture and capture fisheries [43], Nile tilapia has been widely introduced outside its natural distribution range [45] and is cultured globally in sub-tropical and tropical regions [67, 68].

Its large natural distribution area in sub-Saharan Africa and its broad ecological tolerance makes Nile tilapia a successful species in a wide range of aquatic habitats [54]. On a macro-biogeographic scale, molecular studies have revealed clear genetic differentiation and population structure throughout its natural range [7, 36, 59, 60]. According to Bezault et al. [7] three major lineages of Nile tilapia are found in Africa, corresponding to (1) the Ethiopian Rift Valley (primarily the endorheic Awash River drainage), (2) the broadly defined Nilotic region (including the northern part of the Kenyan Rift Valley), and (3) the Sudano-Sahelian region in West Africa. These phylogeographic patterns are largely congruent with paleo-hydrological connectivity and major ichthyofaunal regions [47, 55, 65].

While most previous molecular studies [2, 7, 36, 53] provided data from wide geographical areas across Africa, a more detailed investigations on genetic structure with focus on East Africa confirmed that the Nilotic region and the Ethiopian Rift Valley including adjunct waterbodies harbour several populations with rather high level of potential genetic differentiation [59, 60]. In particular in Ethiopia substantial phylogeographic structure of Nile tilapia had been suggested by studies that include morphological observations [25, 62]. A strong influence by the complex geological and hydrogeographic history of Ethiopia had been concluded, similarly to patterns that have recently been observed for the *Labeobarbus intermedius* complex [5] and small smilogastrin barbs [17]. These studies stress the importance of paleo-geographic, climatic and tectonic events for the ichthyofauna of the region.

Nile tilapia is native to most drainage systems of Ethiopia, but absent from the Wabe Shebelle and Genale-Dawa rivers, where the Sabaki tilapia (*O. spilurus* (Günther 1894)) is found [25, 26, 29]. Four taxa, considered valid species/subspecies or synonymized with Nile tilapia by different authors [25, 62], were originally described from Ethiopia under the following available names (drainage of type localities in parenthesis): *Tilapia cancellata* Nichols, 1923 (presumably Awash); *T. calciati* Gianferrari, 1924 (Atbara-Tekeze); *O. n. filoa* [62] (Awash),and *O. n. tana* [53] (Blue Nile). So far, however, no consistent opinion on taxonomy and delimitation of local Nile tilapia populations has been reached and *O. niloticus* might be a species complex awaiting taxonomic revision [53].

The natural phylogeographic structure of Nile tilapia might be altered as human induced translocations of different Nile tilapia strains for aquaculture and fisheries constitute a major concern to the genetic diversity and integrity of native populations throughout Africa [54, 60]. Besides evidence for strong genetic structure in natural populations [36], signs of admixture due to human induced translocations of non-native stocks have been documented in the upper Nile River drainage [58, 60]. The potential threat of deliberate and uncontrolled introduction of non-native species and/or strains is well known for many freshwater fish species around the world (e.g. [4, 33, 64]). In the case of Nile tilapia, this may pose considerable conservation concerns, as not only admixture between stocks of Nile tilapia but also hybridization with congeneric tilapiine species has been reported [15, 54].

In this study we report a detailed investigation of Nile tilapia populations including the major waterbodies in Ethiopia. Using a dense dataset of 37 Microsatellite loci and including SNP information to define alleles, detailed profiles of genetic variability and differentiation between populations within and between drainage systems, including natural and stocked populations are outlined. We show that not only considerable subpopulation structure exists within one drainage system, but also that Ethiopia harbours several distinct lineages of this species. The data provides essential information to understand the contemporary and historical factors shaping population structure of this species and can be used for informed management decisions. Implications for conservation and biodiversity are discussed.

## Results

### Genetic diversity and HWE deviations between and within drainage systems

A total of 706 alleles was found in the studied samples of Nile tilapia across 37 microsatellite loci. Among the 16 populations investigated, 385 private alleles were observed with the highest number in the Omo-Turkana system (Lake Turkana 224, Gilgel Gibe 19) and Lake Tana (41). Populations in the MER (Main Ethiopian Rift) showed comparably few private alleles with highest numbers in the SMER (Table 1). For all diversity measures, Lake Turkana was more diverse than other populations, while fish from Lake Hayq consistently showed the lowest diversity. Populations of the CMER (lakes Hawassa, Langano and Ziway) appear to have lower genetic variability compared to populations sampled from SMER (lakes Abaya and Chamo) and the Omo-Turkana system. Non-native Nile tilapia populations in lakes Hora, Hayq and Hashenge, and the presumably native population in Lake Metahara generally had low genetic diversity compared to native populations and revealed no private alleles.

**Table 1:** Genetic diversity indices (values are given as mean ± SE) of Nile tilapia (*Oreochromis niloticus*) populations in Ethiopia

Drainage	Population code	*N*	*N*_*a*_	*N*_*e*_	*N*_*p*_	*I*	*H*_*o*_	*H*_*e*_	*uH*_*e*_	*F*	%dHWE	%NA	%poly
Blue Nile	TA	15.19	3.78 ± 0.38	1.97 ± 0.25	1.11 ± 0.34	0.67 ± 0.09	0.23 ± 0.05	0.32 ± 0.04	0.33 ± 0.05	0.47 ± 0.07	64.86	56.76	97.3
	FI	12.87	2.23 ± 0.23	1.71 ± 0.15	0.00 ± 0.00	0.49 ± 0.08	0.31 ± 0.06	0.29 ± 0.05	0.30 ± 0.05	− 0.04 ± 0.06	10.81	10.81	56.76
Omo-Turkana	GG	29.03	3.49 ± 0.32	2.10 ± 0.16	0.51 ± 0.17	0.75 ± 0.08	0.35 ± 0.04	0.42 ± 0.04	0.42 ± 0.04	0.15 ± 0.05	24.32	21.62	94.59
	TU	34.35	10.57 ± 1.05	4.77 ± 0.61	6.05 ± 0.82	1.56 ± 0.13	0.58 ± 0.04	0.64 ± 0.04	0.65 ± 0.04	0.08 ± 0.03	27.03	13.51	100
SMER	AB	19.7	5.08 ± 0.60	2.87 ± 0.38	0.76 ± 0.15	0.98 ± 0.12	0.48 ± 0.05	0.47 ± 0.05	0.48 ± 0.05	− 0.02 ± 0.03	10.81	5.41	91.89
	CH	20.97	4.51 ± 0.54	2.60 ± 0.33	0.51 ± 0.13	0.90 ± 0.11	0.38 ± 0.05	0.44 ± 0.05	0.45 ± 0.05	0.12 ± 0.05	24.32	18.92	83.78
CMER	HW	14.87	2.76 ± 0.31	1.78 ± 0.16	0.16 ± 0.06	0.54 ± 0.09	0.31 ± 0.05	0.30 ± 0.05	0.31 ± 0.05	− 0.01 ± 0.06	13.51	10.81	70.27
	LA	9.84	2.51 ± 0.29	1.75 ± 0.16	0.05 ± 0.04	0.53 ± 0.08	0.32 ± 0.06	0.30 ± 0.05	0.31 ± 0.05	− 0.06 ± 0.07	18.92	8.11	70.27
	ZI	35.35	4.32 ± 0.50	2.04 ± 0.27	0.81 ± 0.17	0.67 ± 0.10	0.36 ± 0.05	0.34 ± 0.05	0.34 ± 0.05	− 0.03 ± 0.04	21.62	2.70	89.19
NMER	KO	24.19	2.30 ± 0.22	1.65 ± 0.12	0.03 ± 0.3	0.49 ± 0.08	0.31 ± 0.05	0.28 ± 0.04	0.29 ± 0.05	− 0.07 ± 0.04	10.81	2.70	62.16
	MT	9.95	2.38 ± 0.27	1.86 ± 0.19	0.00 ± 0.00	0.54 ± 0.09	0.34 ± 0.06	0.31 ± 0.05	0.33 ± 0.05	− 0.08 ± 0.06	5.41	5.41	64.86
	YA	8.6	2.32 ± 0.28	1.68 ± 0.19	0.11 ± 0.05	0.46 ± 0.09	0.30 ± 0.06	0.26 ± 0.04	0.27 ± 0.05	− 0.12 ± 0.07	8.11	8.11	62.16
	KA	9.92	3.68 ± 0.37	2.20 ± 0.26	0.16 ± 0.07	0.80 ± 0.09	0.34 ± 0.06	0.41 ± 0.04	0.43 ± 0.04	0.36 ± 0.08	75.68	45.95	97.3
Endorheic crater lakes	HR	33.35	2.24 ± 0.25	1.69 ± 0.13	0.00 ± 0.00	0.491 ± 0.08	0.29 ± 0.05	0.29 ± 0.05	0.30 ± 0.05	0.03 ± 0.06	16.22	10.81	64.86
	HS	30.97	2.95 ± 0.23	1.59 ± 0.13	0.14 ± 0.06	0.48 ± 0.08	0.28 ± 0.05	0.26 ± 0.04	0.26 ± 0.04	− 0.07 ± 0.05	18.92	5.41	81.08
	HQ	23.24	1.62 ± 0.15	1.32 ± 0.09	0.00 ± 0.00	0.26 ± 0.06	0.19 ± 0.05	0.16 ± 0.04	0.17 ± 0.04	− 0.15 ± 0.06	5.41	0.00	40.54

Average number of individuals per locus (*N*), average number of alleles per locus (*N*_*a*_), number of effective alleles (*N*_*e*_), average number of private alleles (*N*_*p*_), Shannon’s information index (*I*), observed heterozygosity (*H*_*o*_), expected heterozygosity (gene diversity) (*H*_*e*_), unbiased heterozygosity (*uH*_*e*_), inbreeding coefficient (*F*), percentage of loci deviating from HWE (%dHWE), percentage of loci shown a proportion of null alleles above 0.1 (%NA),percentage of polymorphic loci (%poly). Abbreviation of population code is given in Table 3

Only for the populations from Lake Tana and the Awash River at Kada Bada most loci deviated from HWE (Table 1). This was a consequence of an excess of homozygotes which is shown by high values of inbreeding coefficient (*F* = 0.46 for lake Tana and *F* = 0.36 for Kada Bada). These populations were also the ones showing more loci with proportion of null alleles above 0.1 (Table 1).

### Population differentiation and hierarchical clustering

Pairwise genetic differentiation (*F*_*ST*_ values) among the 16 Nile tilapia populations investigated are given in Table 2. We considered *F*_*ST*_ values above 0.40 as high, between 0.20 and 0.39 as medium and below 0.20 as low. In general, the highest *F*_*ST*_ values were found in comparisons involving populations from Lake Tana (0.38–0.56) and Gilgel Gibe (0.30–0.47). Though, the Gilgel Gibe population showed relatively low *F*_*ST*_ values with Lake Turkana that is from the same drainage system. Populations from the SMER were distinct from Lake Turkana (0.19–0.22) while genetically closer to the CMER and the NMER populations. While the interconnected CMER lakes showed low *F*_*ST*_ values (0.02–0.04), a higher differentiation was found in the Awash drainage (0.05–0.13) with highest *F*_*ST*_ values between Lake Metahara and Lower Awash (Yardi).

**Table 2:** The pairwise population FST values of 16 Nile tilapia (*Oreochromis niloticus*) populations in Ethiopia

Ta	Fi	Gg	Tu	Ab	Ch	Hw	La	Zi	Ko	Mt	Ya	Ka	Hr	Hs	Hq	
0																TA
0.51	0															FI
0.38	0.42	0														GG
0.29	0.31	0.13	0													TU
0.41	0.12	0.3	0.19	0												AB
0.42	0.15	0.32	0.22	0.04	0											CH
0.52	0.11	0.42	0.3	0.09	0.12	0										HW
0.51	0.09	0.41	0.3	0.1	0.13	0.04	0									LA
0.5	0.07	0.4	0.28	0.08	0.1	0.03	0.02	0								ZI
0.52	0.06	0.44	0.31	0.11	0.14	0.08	0.07	0.05	0							KO
0.5	0.05	0.4	0.29	0.13	0.16	0.13	0.11	0.09	0.1	0						MT
0.53	0.13	0.42	0.31	0.12	0.15	0.09	0.07	0.05	0.1	0.13	0					YA
0.41	0.08	0.35	0.25	0.08	0.11	0.06	0.06	0.04	0.07	0.09	0.05	0				KA
0.51	0.06	0.42	0.31	0.15	0.18	0.13	0.13	0.1	0.1	0.04	0.13	0.09	0			HR
0.5	0.11	0.42	0.3	0.18	0.2	0.2	0.18	0.16	0.19	0.06	0.2	0.14	0.03	0		HS
0.56	0.19	0.47	0.35	0.22	0.26	0.26	0.24	0.19	0.27	0.1	0.25	0.17	0.1	0.08	0	HQ

Abbreviation of population code is given in Table 3. FST values above 0.40 we considered high, between 0.20 and 0.39 medium and below 0.20 low

The analysis of the distribution of genetic variation (AMOVA) indicated that 38% of variation was explained by differences among populations, and 53% among individuals within populations. The remaining 9% of variation were attributed to differences within individual.

**Fig. 1:**
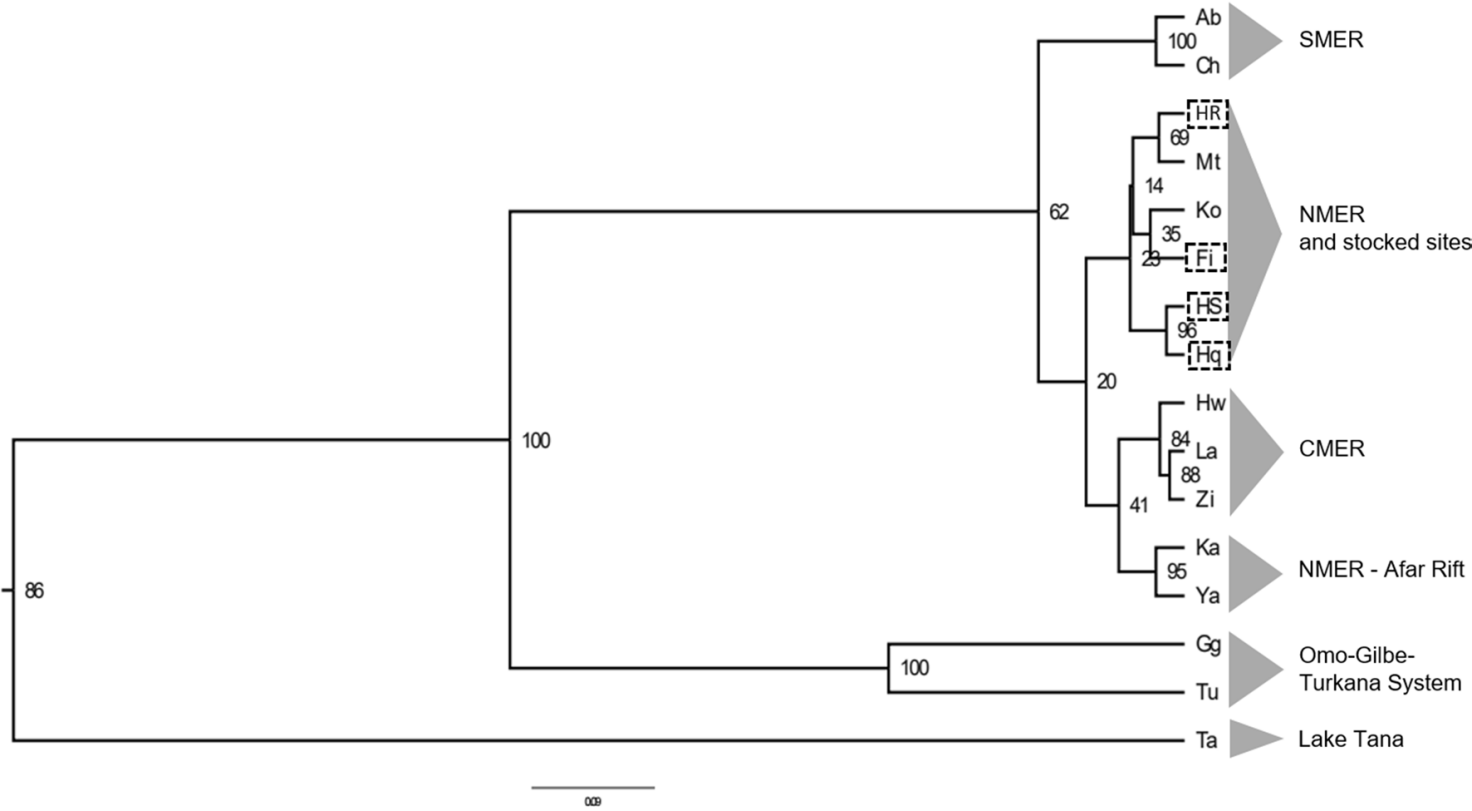
UPGMA dendrogram of 16 populations of Nile tilapia from Ethiopia, constructed using Nei’s genetic distance. Dashed boxes indicate stocked sites. Abbreviation of sampling sites as given in Table 3. Support is given by bootstrap values

Genetic distances between populations varied from 0.012 (between Kada Bada and Lake Yardi in the Awash drainage) to 1.981 (between Hawassa and Lake Tana) (Additional file 1: Table S1), as illustrated in the UPGMA (Fig. 1). The population from Lake Tana was the most divergent, followed by a well-supported (100%) group composed of the Omo-Turkana drainage. Among the MER, populations from the southern part (lakes Chamo and Abaya) are distinct from the CMER and NMER lakes forming a supported cluster (100%). The SMER populations are distinguished from the Lake Turkana and Gilgel Gibe by relatively high *F*_*ST*_ values (0.19–0.32). While populations from lakes in the CMER cluster together with samples from the Lower Awash River (Yardi and Kada Bada), they appear to be distinct from the geographically closely situated Koka and Metahara populations. Though bootstrap support is low, the latter were found to cluster together with stocked fish from crater Lake Hora and the Fincha Reservoir in the Blue Nile drainage.

**Fig. 2:**
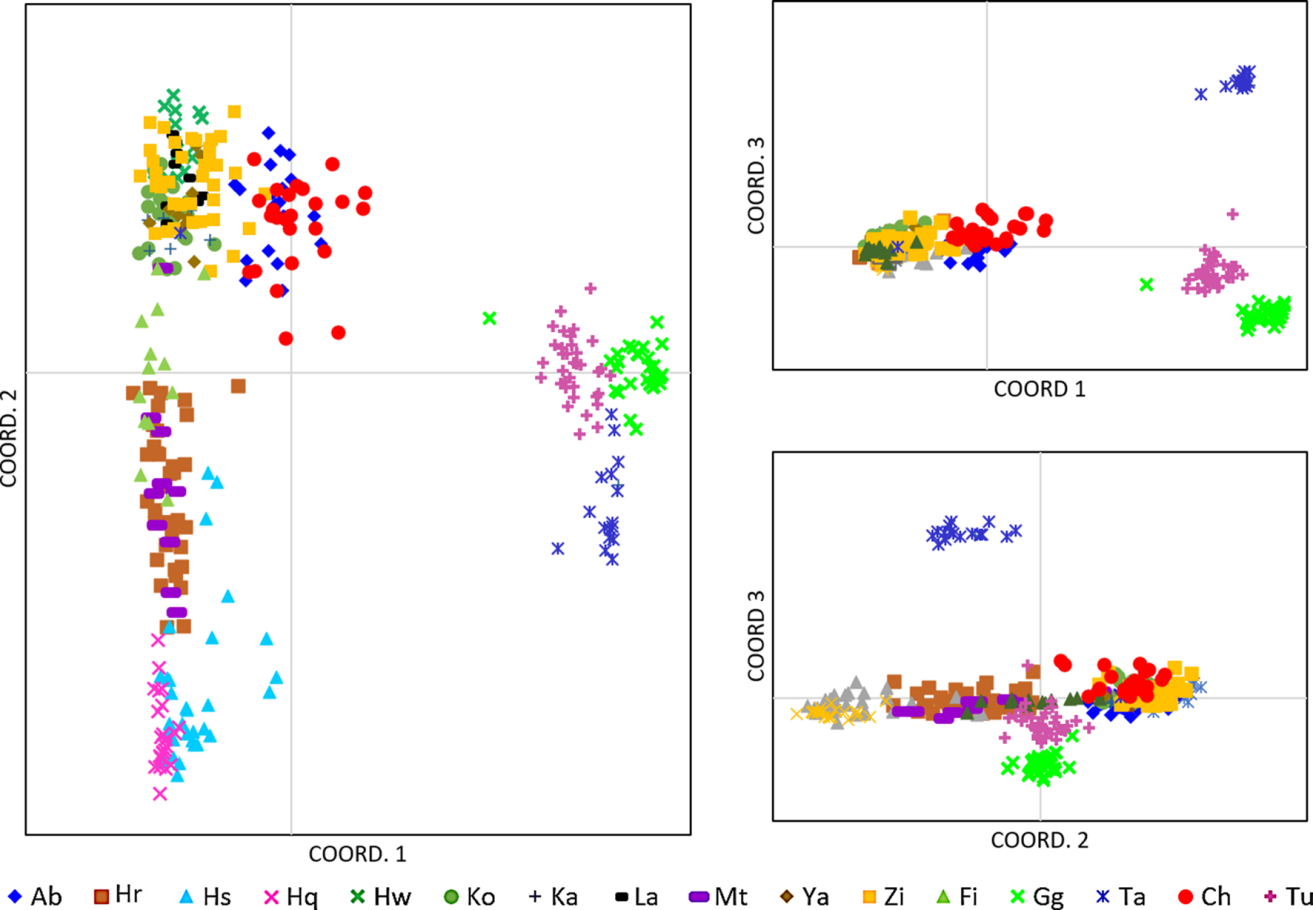
Principal Coordinate Analysis (PCoA) plots illustrating genetic similarity of Nile tilapia populations in Ethiopia considering all populations. The first axis explains 26.2%, second axis 10% and third axis 7.2% of the variation, accumulating to 43.3%. Abbreviation of sampling sites as given in Table 3

**Fig. 3:**
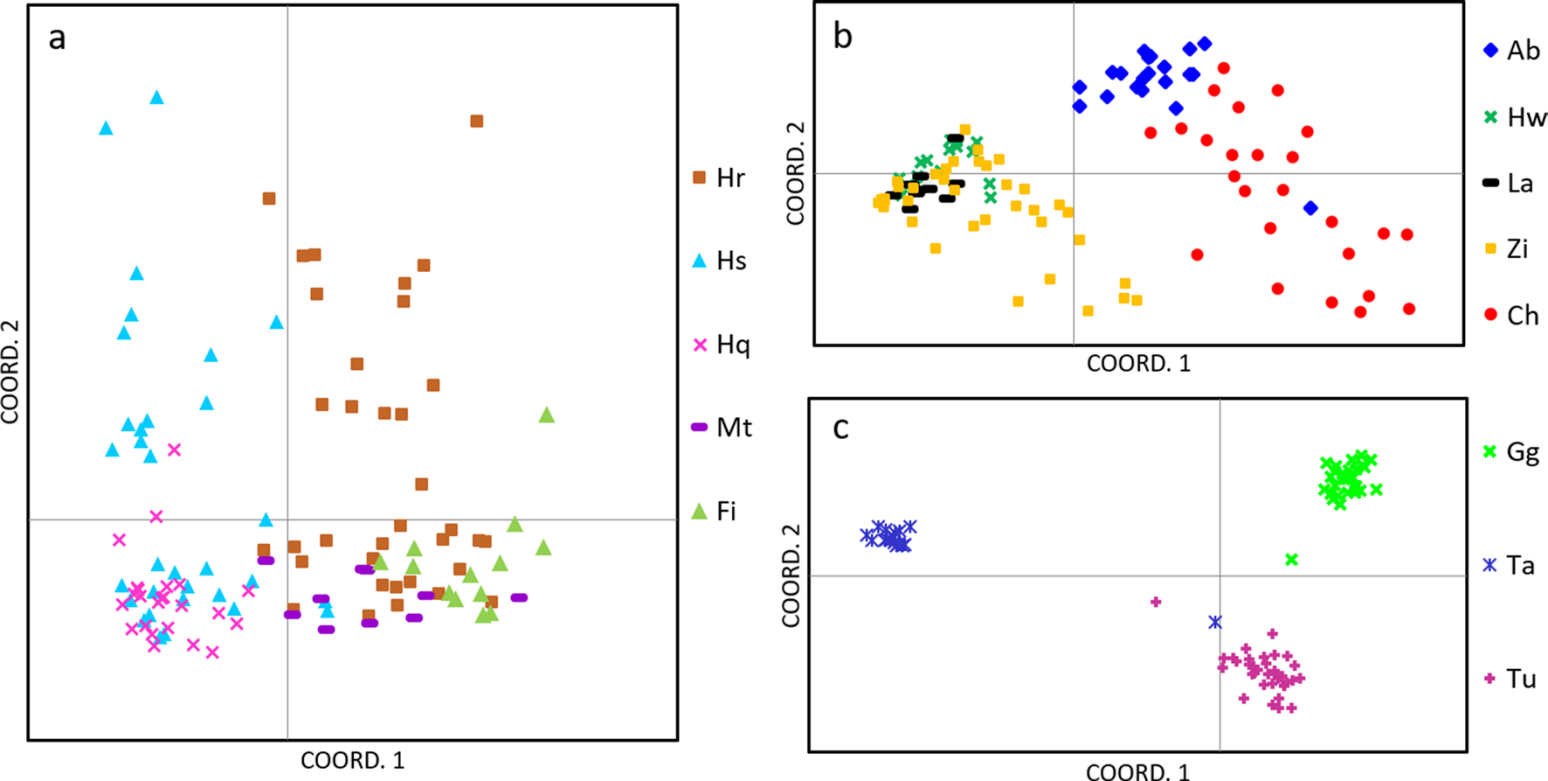
Principal Coordinate Analysis (PCoA) plots illustrating genetic similarity of Nile tilapia populations considering the following subgroups: **a** translocated/stocked populations, **b** populations from the Southern and Central Rift Valley Lakes, **c** most divergent populations from Omo-Turkana (Gilgel Gibe, Turkana) and Lake Tana. Abbreviation of sampling sites as given in Table 3

PCoA analysis showed a similar segregation pattern (Figs. 2 and 3). The analysis of all 16 populations revealed a clear distinction between Lake Turkana, Gilgel Gibe and Lake Tana from all other populations investigated along the first coordinate. Along the second and third coordinates Lake Tana population is strongly separated, so the three distinct groups as indicated in the UPGMA are supported (Fig. 2). One specimen of Lake Tana groups closer to the rift valley lakes. Populations from the MER cluster together, but fish from the SMER are clearly distinct forming a separate cluster without any overlap with samples from CMER and NMER. Stocked populations from the Blue Nile drainage and the crater lakes form an indistinct group with some overlap to the MER populations. Substantial overlap was observed between populations in lakes Hashenge and Hayq as well as between lakes Metahara, Hora and the Fincha Reservoir (Fig. 3).

### Genetic structure and signs of admixture

**Fig. 4:**
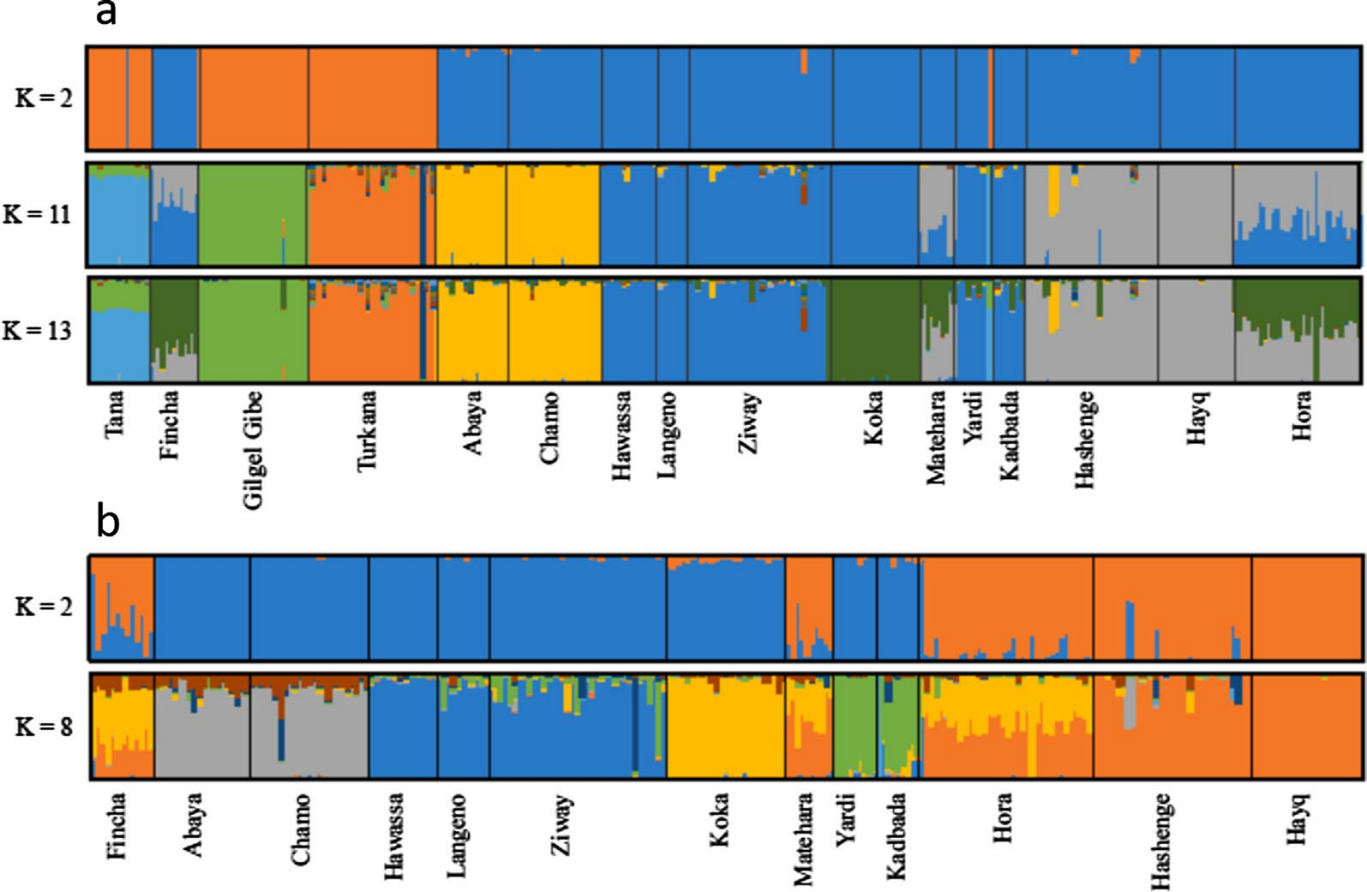
STRUCTURE analysis (admixture model) for **a** all Nile tilapia samples investigated (optimal cluster K = 2, suboptimal clusters K = 11 and K = 13) and **b** excluding the most divergent populations from Omo-Turkana (Gilgel Gibe, Turkana) and Lake Tana (optimal cluster K = 2, suboptimal clusters K = 8). Each bar representing a single individual, and each colour representing proportion of membership (population) with regard to each genetic cluster

Based on delta K (ΔK) values K = 2 was considered the best-fitted to the data. STRUCTURE HARVESTER results also suggested K = 11 and K = 13 as the second and third-best fit to the data. Plots for these K values are shown in Fig. 4a while the result for all K values up to 13 are in Additional file 2: Figure S1. For K = 2, the individuals from Gilgel Gibe, Tana and Turkana were assigned to one genetic cluster (orange) and the remaining individuals to the other cluster (blue). Some individuals showed mixed assignment to both clusters or were assigned to an different cluster then the other members of their population. For K = 11 (Fig. 4a) individual fish from SMER sub-basin appeared to share the same genetic cluster, while individuals from the CMER, Awash system and Fincha formed a separate cluster. Clustering is congruent with the UPGMA dendrogram and PCoA and reflects the defined drainages. The populations from Turkana, Gilgel Gibe, and Tana were assigned to a different cluster from the remaining populations for all K values. The populations from the CMER and NMER were assigned to the same cluster (blue) for all K values. The SMER cluster together (yellow cluster) for K = 11 and K = 13. For K = 13 the NMER sites and stocked populations are assigned to two clusters being one (green) more prevalent in Koka and the other (grey) in Hashenge and Hayq. The remaining populations from this group shows several degrees of mixed assignment to these two clusters. Some other evidences of admixture were found in other populations. For example, two individuals from Hashenge that show some degree of assignment to the SMER cluster. Similarly, to the PCoA the same individuals from Turkana and Tana are assigned to clusters from other populations. When excluding the most divergent populations based on *F*_*ST*_ and Nei distance analysis (Turkana, Gilgel Gibe, and Tana) the best K was 2 and suboptimal was 8. The result obtained did not change from the analysis including all samples (Fig. 4b). The result for all K values up to 8 are in Additional file 3: Figure S2.

## Discussion

Nile tilapia shows a very pronounced genetic structure in its native range that is on one hand strongly correlated with historical connectivity between waterbodies but also influenced by contemporary stocking activities. This underlines the potential of translocations causing genetic changes, loss of variation, loss of specific adaptations and changes in genetic structure in accordance to Laikre et al. [34]. Nile tilapia population structure is highly congruent with the respective drainage basins a structure which is only intercepted by the stocked populations. In earlier studies with the same marker system implemented here [59, 60], where only a few representatives of Ethiopian Nile tilapia were also included, five well supported clusters were identified. One contained the West African populations, while the remaining four groups corresponded to the East African populations of: Uganda, the Kenyan Rift Valley (namely Lake Turkana), Ethiopian Rift Valley and Lake Tana. The level of differentiation in East Africa was at a similar height to the one found between East and West Africa. Our study now, adds a detailed view on genetic structure of Ethiopian samples: three major clusters divided by high *F*_*ST*_ values exist in Ethiopia that correspond to the flowing groups from Tibihika et al. [59, 60]: (1) Lake Tana, (2) the Kenyan Rift Valley (Omo-Turkana system), and (3) the Ethiopian Rift Valley populations. Further divisions of these groups were supported by the geographical congruence of the patterns showed by the UPGMA, and STRUCTURE analysis for higher K values. In this context, some genetic structure was found within some drainages, indicating that there is limited geneflow between most waterbodies.

### Genetic diversity and differentiation between and within drainages

Genetic diversity, indicated by the mean number of alleles (*N*_*a*_) and heterozygosity, of Ethiopian Nile tilapia populations was slightly lower than in the analysis of East African populations [60]. Genetic diversity parameters estimated here were also lower than the ones from other African Nile tilapia populations using other marker systems [7, 21, 41]. Regardless, finding together with the observation of subpopulation structure within the same drainage basins is in accordance with the idea that the high geographic fragmentation of Ethiopian water bodies contributes to a low connectivity between Nile Tilapia populations [7].

Pairewise *F*_*ST*_ values varied between the 16 populations, ranging from 0.02 to 0.69 (Table 2). Most of the values were greater than 0.25, indicating a high level of genetic differentiation among them. The larger values suggest that these populations may have evolved in isolation and in some cases may reflect different taxa. This may be the case of the populations Gilgle Gibe, Tana and Turkana that display the highest *F*_*ST*_ values when compared to others, and cluster apart from the Ethiopian sites for all analyses. Given the structure pattern found three potential taxonomic groups may be defined: (1) Lake Tana, (2) Turkana and Gilge Gibe, and (3) Ethiopian Rift Valley populations. Tibihika et al. [60] had already shown similar groups when comparing some Ethiopian populations (Tana, Ziway, Hashenge, Chamo) and lake Turkana with Ugandan populations. In this case, Lake Turkana samples are genetically more similar to the Ugandan populations and most likely represent the same species. The MER populations (Ziway, Hashenge, Chamo) were more divergent when compared to Uganda than for example West African populations indicating that they may represent a different taxon. The same applies to Lake Tana.

The taxonomic revision of Nile Tilapia has been suggested by other authors. Trewavas [62], Seyoum and Kornfield [53] and Tibihika et al. [60], found similar patterns which led them to recommend a taxonomic revision for these populations. Bezault et al. [7] also showed the importance of different paleo-geographic and climatic events shaping the genetic structure of distinct populations. Lake Tana passed through several low and high-water level events that might also contributed to its unique fish fauna and unique Nile tilapia genetic structure. Previously, based on morphological examination, Trewavas [62] treated the population from this lake as sub-species *O. n. cancellatus*, which was later described as *O. n. tana* by Seyoum and Kornfield [53]. However, Agnèse et al. [2] suggested that these results should be considered with reservation.

Turkana and Gilgel Gibe were clustered together and belong to the Turkana system (Figs. 1 and 2). The Gilgel Gibe population was sampled from a dam built on the Gilgel Gibe, one of the large tributaries to the Omo River, which is the main river flowing into Lake Turkana, explaining the similarity between these populations. Apart from having the highest N_a_, Turkana also exhibited the highest mean number of private alleles pre locus (Table 1). The same pattern was found when compared with Ugandan populations [60].

The CMER lakes (Ziway, Langano, and Hawassa) and the Awash (Kadbada and Yardi) populations were genetically more similar to each other than to the SMER populations, Abaya and Chamo. This is also shown by Nei’s genetic distances (Additional file 1: Table S1) that indicated the lowes values for the comparison between Langano and Ziway populations. Distances and clustering clustering patterns precisely reflect the sub-regions of the Main Ethiopian Rift, which is traditionally divided into the Southern (Lakes Abaya and Chamo), the Central (Lakes Hawassa, Langano, and Ziway) and the Northern (Yardi and Kadbada) Ethiopian Rift Valley including the Awash River systems (see [1, 8, 17], and references therein).

Sagri et al. [49] and Benvenuti and Carnicelli [6] documented the connections between the Upper Awash drainage and the lakes in the CMER, which may explain the similarity of the populations in these water bodies. Additionally, the relatively low to moderate genetic variability in these lakes (Table 1) may be attributed to factors such as habitat destruction and high fishing pressure that fish populations in these water bodies are experiencing [66]. The remaining two populations in the NMER, Yardi and Kadbada are geographically close and fed by the Awash River which may facilitate gene-flow between them.

In contrast lakes Chamo and Abaya in the SMER are different from the other Rift valley lakes in their fish fauna, exhibiting Nilo-Sudanic affinities due to their historic connection to the Turkana and White Nile systems [28]. This study also showed a clear evidence about water courses connecting of the Chamo-Abaya, Chew Bahir, Turkana and White Nile systems during the late Pleistocene-early Holocene [20]. Interestingly, despite these ichthyofaunal affinities, the current study did not reveal strong evidence of genetic similarity between the Lake Turkana and the SMER populations of Nile tilapia.

The high genetic variability in lake Abay and Chamo could be explained by their relatively large size (compare Table 3), as in larger Lakes niches are more variable than the smaller ones, creating suitable conditions for higher diversity [38]. Bezault et al. [7] suggested that environmental factors such as habitat heterogeneity and intrinsic factors (such as habitat preference) have an impact on the gene pool at intra-population level.

**Table 3:** Sampling sites and population information

Drainage	Sampling site	Population code	Status of population	Coordinates	Altitude (m a.s.l.)	Area (km^2^)	Sample size
Blue Nile	Lake Tana	Ta	Native	11° 58′ N, 37° 18′ E	1788	3500	17
	Fincha Reservoir	F	Introduced	9° 32′ N, 37°14′ E	2226	1318	13
Omo-Turkana	Gilgel Gibe Reservoir	Gg	Native	7°47′N, 37°17′E	1,650	62	30
	Lake Turkana	Tu	Native	4° 32′ N, 36° 8′ E	365	6405	35
SMER	Lake Abaya	Ab	Native	6° 15′ N, 37° 55′ E	1177	1162	20
	Lake Chamo	Ch	Native	5° 50′ N, 37° 35′ E	1110	317	25
CMER	Lake Hawassa [Awassa]	Hw	Native	7° 03′ N, 38° 26′ E	1686	129	15
	Lake Langano	L	Native	7° 35′ N, 38° 45′ E	1582	241	10
	Lake Ziway	Zi	Native	8° 00′ N, 38° 50′ E	1636	442	37
NMER	Koka Reservoir	Ko	Native and introduced	8° 25′ N, 39° 04′ E	1592	255	25
	Lake Metahara [Beseka]	Mt	Native and introduced	8° 54′ N, 39° 53′ E	954	43	10
	Lake Yardi	Ya	Native	10° 14′ N, 40° 32′ E	565	93	9
	Awash River at Kada Bada	Ka	native	10° 13′ N, 40°34′E	565	–	10
Endorheic crater lakes	Lake Hora	Hr	Introduced	8° 45′ N, 38° 59′ E	1875	1.03	35
	Lake Hashenge [Ashenge]	Hs	Introduced	12° 34′ N, 39° 30′ E	2442	20	33
	Lake Hayq [Hayk]	Hq	Introduced	11° 20′ N, 39°43′E	1911	23	24

*SMER* Southern Main Ethiopian Rift, *CMER* Central Main Ethiopian Rift, *NMER* Northern Main Ethiopian Rift

Only two populations deviated from HWE, Tana and the Awash River at Kada Bada. A possible explanation might be Wallon effect given that both habitats are heterogeneous and show mixed assignment to multiple clusters. However, a different explanation can be given, especially for the deviations found for Lake Tana, since this population was the one showing the most loci with high proportion of null alleles. This lake is the most divergent population and it is suspected to be a different species. Using microsatellite markers for cross species amplification can lead to an excess of null alleles due to mutations at the primer-binding site which contribute to deviations from the HWE [63]. Thus, if Lake Turkana is in fact another species the HWE deviations are not surprising.

### Translocated and stocked populations

Hayq, Hashenge, Fincha, Koka, Metahara, and Hora, which include the 4 non-native populations, are grouped together by the UGPMA (Fig. 1) and PCoA (Figs. 2 and 3). This was partly expected, as the stocked Hora population, with an unknown origin, was also the source for stocking activities in Hayq, Hashenge [61], and Fincha [56] by a limited number of fish. Additionally, Bezault et al. [7] reported low genetic diversity based on both allelic richness and heterozygosity for Nile tilapia populations in Lake Hora. Therefore, their initial diversity was probably already reduced due to a founder effect caused by stoking activities [50]. Furthermore, these lakes are also small in size and Harrisson et al. [30] noted that genetic diversity deteriorates faster in small and isolated populations due to genetic drift, which can lead to the loss or reduction in adaptive potential and fitness, and an increase in inbreeding (accumulation of genetic load). Moreover, mass fish kills have been reported in Lake Hayq [31] further reducing the effective population size. All this may contribute to the low diversity observed for the Nile tilapia population of Lake Hayq and the possibility that this population may suffer from inbreeding depression needs further investigation. The genetic similarities of the stocked populations with the individuals from Metahara suggest that either this could be the original source of stocking material, or both have been stocked from an unknown origin. The native populations of Koka and Metahara have been also influenced by stocking activities ([61] and references therein), explaining the proximity within this group. As mentioned above, the Fincha population has been stocked in late 1970s to fill an empty pelagic niche and to provide cheap protein to the local communities [56, 61]. Despite the physical connectivity between Fincha and Tana through the Nile River, these water bodies did not exhibit genetic similarity. The strong rapids and falls (up to 40 m high) present in the Nile (Abay) River [40], might have also created a natural barrier to gene flow between the populations which maintains them apart. None of the populations with low genetic diversity deviated from HWE and therefore if there is a genetic diversity lost this corresponds to either a slow continuous process or to a past event [10].

### Threats, outlooks, conservation and management implications

Deforestation, habitat degradation, and overfishing were identified as major threats to natural fish population in sub-Saharan Africa and the same applies to Ethiopia (e.g. [23, 37, 66]). Pollution and lake level changes will exacerbate the reduction of populations sizes and thus speed genetic drift effect further reducing genetic diversity of fish populations [23]. Moreover, some of the lakes are small in size (e.g., lakes Hora and Hashenge) and therefore might suffer heavily from the effects of anthropogenic activities and changing climatic conditions. These factors may also reduce the effective population size in these lakes which will lead to inbreeding depression reducing adaptive potential [11, 30]. Furthermore, with the growth of the human population in Ethiopia, an increasing demand for protein and fish [43] will ultimately lead to the translocation of commercially important fish species to different parts of the country, additional supported by the creation of artificial water bodies for various purposes. There is a high possibility that some of these stocks will end up in the ranges of the native population and thereby deteriorating this unique genetic resource.

Based on the high genetic differentiation between some of these populations it is expected that uncontrolled translocations may lead to catastrophic consequences such as out breeding depression [48]. This will not only threaten local catch fisheries but also corrupt the seed populations for aquaculture. Thus, the results of this study bring up the opportunity to develop local Nile tilapia strains for a sustainable aquaculture [13]. According to the genetic structure results, drainage specific strains should be established. Moreover, their use outside their region of origin should be prevented since aquaculture escapees may contribute to unwanted geneflow between drainages.

The development of a sustainable aquaculture practice could offer alternative livelihoods and help fish stocks in natural ecosystems to recover, thereby easing overfishing pressures [51]. Sustainable utilization of fisheries resources requires informed management strategies. Our study shows low level of genetic diversity that can be affected by overexploitation [3]. Additionally, hydropower projects also effects the genetic diversity and integrity of fish populations [11]. The implementation of environmental safety standards need to consider all these factors and activities to reduce contamination and loss of the unique genetic resources in the country.

## Conclusion

In this study, we clearly showed that the genetic structure of Nile tilapia populations in Ethiopia is complex. The genetic structure patterns found here are likely a consequence of both biogeographic and anthropogenic factors. Our results indicate that the Abaya, Chamo, Gilgel Gibe and Turkana populations contain the highest genetic variability while the translocated populations of Hora, Hashenge and Hayq showed the least. High *F*_*ST*_ values between the populations indicate a high level of genetic differentiation among these 16 populations. It also suggested that many of the assessed populations are genetically different and this indicates that they may have evolved in isolation. Genetic clustering of the native populations reflect their geographic distribution pattern for the Main Ethiopian Rift, the Omo-Turkana system and Lake Tana. Moreover, the large genetic differentiation from Gilgel Gibe, Tana and Turkana, indicates greater diversity and the possible existence of multiple sub-species or even species. Nevertheless, the taxonomic position of Nile tilapia in these water bodies should be further investigated using mtDNA analysis. The results presented herein have important implications concerning anthropogenic activities, such as stocking programs and aquaculture practices, for securing the genetic resources in the country and advancing our understanding of biodiversity, phylogeny, evolution and development towards phylogenetically more accurate taxonomic classifications.

## Materials and Methods

### Sample collection and study locations

**Fig. 5:**
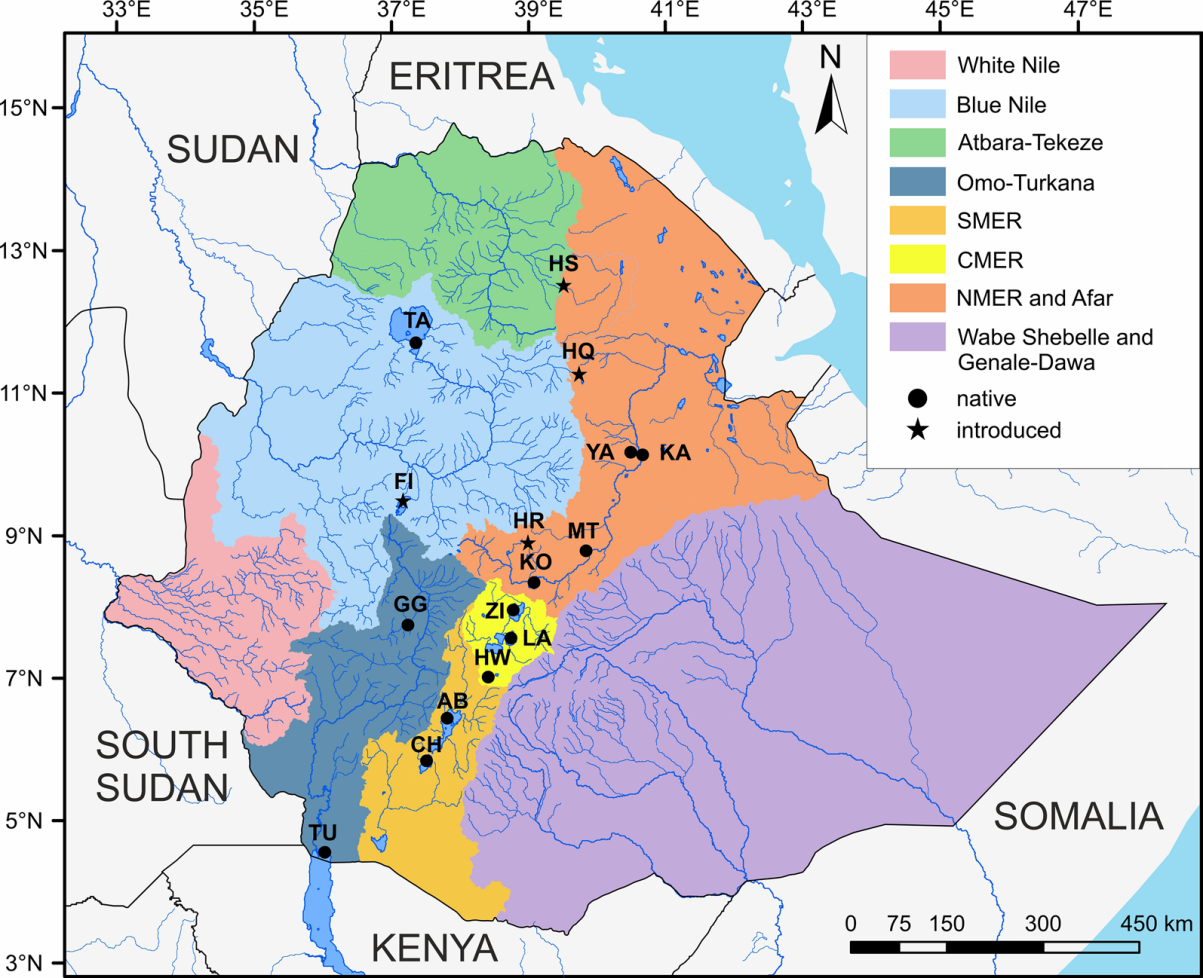
Map of the study areas and major drainage systems in Ethiopia as described by Golubtsov and Darkov [27]. Abbreviation of sampling sites as given in Table 3

A total of 348 individuals of Nile tilapia were sampled from 16 localities across different drainage systems of Ethiopia between 2017 and 2019 (Table 3, Fig. 5). Samples were collected using gill nets (mesh size ranging between 6 and 12 mm) and seine nets, and purchased from local fishermen. Identification of the sampled fish specimens was done based on various literatures [22, 29, 62]. The fish studied ranged in total length from 5 to 42 cm, the smallest from Yardi and the largest from Lake Chamo. Fresh tissue samples were taken from the pectoral fins and directly preserved in 97% ethanol. Sampling sites were located in the following drainage systems, given that the Blue Nile and Omo-Turkana systems correspond to Bezault et al. [7] Nilotic lineage while the remaining basins to the Ethiopian Rift Valley lineage:

1. Abay/Blue Nile River: Lake Tana (TA) and Fincha Reservoir (FI) represent populations from the Nile drainage. Lake Tana, the largest lake in Ethiopia, is the source of the Blue Nile but largely separated from this river by the Blue Nile Falls. The Fincha Reservoir is located in the headwaters of the Fincha River, a left bank tributary of the Blue Nile. The Nile tilapia population in the reservoir is introduced [14, 61].
2. Omo-Turkana system: Gilgel Gibe Reservoir (GG) and Lake Turkana (TU) represent two natural populations of Nile tilapia in the Omo-Turkana system. The Gilgel Gibe Dam and the associated reservoir are situated along the Omo River, forming one of the largest reservoirs in Ethiopia. Lake Turkana is a large alkaline lake in the northern Kenyan Rift with paleo-connectivity to the Nile [39]. Less than 15% of the lake portion is located in Ethiopia [57].
3. Southern Main Ethiopian Rift (SMER): Lakes Abaya (AB) and Chamo (CH), represent a system of interconnected lakes with a natural population of Nile tilapia and evidence of past connections to lakes Chew Bahir and Turkana [20].
4. Central Main Ethiopian Rift (CMER): Lakes Hawassa (HW), Langano (LA) and Ziway (ZI) represent a system of partially interconnected lakes in the central part of the Main Ethiopian Rift with a natural population of Nile tilapia. Evidence for frequent lake level fluctuations and past connections to the Awash system are known [49].
5. Northern Main Ethiopian Rift (NMER): The Awash River systems with sampling sites in the Koka Reservoir (KO), lakes Metahara (MT) and Yardi (YA) and the mainstem Awash River at Kada Bada (KA), represent presumably natural populations of Nile tilapia. Englmaier et al. [18] recently published a detailed description of the Awash River drainage and its fauna. There are reports of stocking activities in Koka Reservoir and the saline Metahara Lake in addition to the natural population of Nile tilapia ([61] and references therein).
6. Volcanic crater lakes in the NMER: Lake Hora (HR), is one of five crater lakes near the town of Debre Zeyit (Bishoftu). A long history of stocking Nile tilapia from various sources is reported for all five lakes [61].
7. Volcanic crater lakes in the Ethiopia Highlands: Lakes Hashenge (HS) and Hayq (HQ). These lakes are located near the north-western escarpment of the MER at altitudes between 2000 and 2500 m a.s.l. Both lakes were stocked with Nile tilapia of unknown origin [61].

### Genotyping by amplicon sequencing (SSR–GBAS)

Whole genomic DNA was extracted using following the protocol from Tibihika et al. [59] using the magnetic beads from the MagSi-DNA Vegetal kit (MagnaMedics, Geleen, Netherlands). DNA quality was visualised with gel electrophoresis and samples with visible DNA at high molecular weight were further processed. PCR reactions were carried out using 42 microsatellite primers (Additional file 4: Table S2) previously designed and tested to investigate East African Nile tilapia populations [59, 60]. The primers are elongated by motifs allowing for amplicon library preparation according to the TrueSeq chemistry for Illumina. Primer pairs had been arranged into four multiplex mixes as described and indicated earlier [59]. PCR was performed as 10 µl reaction containing 1 µl template (genomic DNA), 0.25 µM of each primer and 5 µl Quiagen Multiplex Mastermix (Qiagen, Netherlands), containing the polymerase, buffer and nucleotides. The thermo-cycler reactions were carried out under the following conditions: initial denaturation at 95 °C for 15 min followed by 30 cycles at 95 °C for 30 s, annealing at 55 °C for 60 s (annealing temperature similar for all primer pairs), elongation at 72 °C for 1 min and a final extension at 72 °C for 10 min. PCR products per sample were pooled, cleaned with AMPure magnetic beads (Beckman Coulter, USA), and 1 µl of the resulting solution was used for a second PCR to introduce the sample specific index combination for amplicon sequencing using Illumina. A detailed description of the procedure is given in Curto et al. [12] and Tibihika et al. [59]. PCR products of all individually indexed samples were pooled and used for a paired-end 300 bp sequencing run on an Illumina MiSeq at the Genomics Service Unit in Ludwig Maximilian Universität, München, Germany.

Sequences generated by Illumina were subsequently quality checked using criteria described in Curto et al. [12]. Allele calling was done using the Phython scripts described in Curto et al. [12]. The scripts use length information to define alleles analogous to traditional microsatellite analysis but also based on their composition. Allele calling is therefore including all sequence information. The scripts are available at https://github.com/mcurto/SSR-GBS-pipeline. For further analyses, all loci and samples with missing genotypes ≥ 50% were excluded [12] leaving a total number of 37 microsatellite markers (Additional file 4: Table S2).

### Statistical analyses

Genetic diversity and variability per population was estimated based on the average number of alleles per locus (*N*_*a*_), effective number of alleles (*N*_*e*_), total/mean number of private alleles (*N*_*p*_), observed heterozygosity (*H*_*o*_), expected heterozygosity (*H*_*e*_), *F*-statistics (*F*_*ST*_) and Shannon’s information index (*I*). Analysis of molecular variance (AMOVA) was applied to partition the total genetic variance into components explaining divergence between populations, among individuals within populations, and among individuals within each sampling site. Moreover, deviation from Hardy Weinberg Equilibrium (HWE) per loci were estimated. These analyses were performed using GenAlEx v6.503 [44]. Proportion of null alleles was estimated with the program FreeNA [9]. Presence of null alleles was considered for proportions above 0.1.

Genetic structure and differentiation between populations was evaluated by calculating *F*_*ST*_ values per population and pairwise Nei’s genetic distances [42]. Genetic distances between populations were visualized as UPGMA as implemented in Populations v.1.2.32 [35]. In this scope support values were estimated by preforming 1000 bootstrap replicates with loci resampling. Principal Coordinate Analysis (PCoA) was calculated with GenAlEx v.6.503 [44].

Population structure was further examined by assigning individuals to populations based on the Bayesian clustering method using STRUCTURE v2.3.4 [24, 46]. This program groups individuals based on their genotypes without a priori delineation of populations. The optimal number of sub-populations (∆*K*) was estimated based on the rate change in the log probability of data between successive *K* values according to the Delta *K* method [19]. Independent runs for *K* values ranging from 1 to 20 with 10 replicates were performed with a burn-in length of 10,000 Markov chain Monte Carlo (MCMC) generations followed by 10,000 generations. This short burn-in length was chosen to save computational time. To evaluate if a longer burn-in was required, besides checking for convergence of FST and alpha parameters, we performed a run with 100,000 generation burn-in which the result was congruent with the short one. Since this was the case burn-in length was not increased. Both Ln (*K*) and delta K (∆*K*) statistics were used to select the most likely number of clusters using STRUCTURE HARVESTER (http://taylor0.biology.ucla.edu/structureHarvester/) [16] which validates multiple K values for maximum detection. Results from multiple replicates were summarized using the online pipeline CLUMPAK (http://clumpak.tau.ac.il/) [32]. By doing so we expect that possible incongruence caused by the short burn-in are diluted.

## Supplementary Information

**Additional file 1: Table S1.** Unbiased Nei’s genetic distance between 16 Nile tilapia populations revealed by 37 microsatellite loci.

**Additional file 2: Figure S1.** Structure analysis (admixture model) for all Nile tilapia samples investigated for all K values up to 13.

**Additional file 3: Figure S2.** Structure analysis (admixture model) excluding the most divergent populations from Omo-Turkana (Gilgel Gibe, Turkana) and Lake Tana for all K values up to 8.

**Additional file 4: Table S2.** Characteristics of microsatellite loci used to genotype Nile tilapia populations in Ethiopia.

## Data Availability

Raw sequence data were submitted to the sequence read archive (SRA) database and can be accessed under the reference numbers SRR13996602 to SRR13996367.
