# Supplementary material for: Genetic diversity of Nile tilapia (Oreochromis niloticus) populations in Ethiopia: insights from nuclear DNA microsatellites and implications for conservation
Source: BMC Ecol Evol. 2021 Jun 7;21:113. doi: 10.1186/s12862-021-01829-2 (PMC8183085; doi:10.1186/s12862-021-01829-2)
Supplement: Supplementary file 1 — Additional file 1: Table S1. Unbiased Nei’s genetic distance between 16 Nile tilapia populations revealed by 37 microsatellite loci. [file 12862_2021_1829_MOESM1_ESM.docx]

Table S1. Unbiased Nei’s genetic distance between 16 Nile tilapia populations revealed by 37 microsatellite loci. Abbreviation of population code is given in Table 3.

| **Ab** | **DZ** | **Hs** | **Hq** | **Hw** | **Ko** | **Ka** | **La** | **Mt** | **Ya** | **Zi** | **Fi** | **Gg** | **Ta** | **Ch** | **Tu** |  |
| --- | --- | --- | --- | --- | --- | --- | --- | --- | --- | --- | --- | --- | --- | --- | --- | --- |
| 0.000 |  |  |  |  |  |  |  |  |  |  |  |  |  |  |  | **Ab** |
| 0.236 | 0.000 |  |  |  |  |  |  |  |  |  |  |  |  |  |  | **DZ** |
| 0.295 | 0.078 | 0.000 |  |  |  |  |  |  |  |  |  |  |  |  |  | **Hs** |
| 0.345 | 0.067 | 0.020 | 0.000 |  |  |  |  |  |  |  |  |  |  |  |  | **Hq** |
| 0.120 | 0.154 | 0.311 | 0.309 | 0.000 |  |  |  |  |  |  |  |  |  |  |  | **Hw** |
| 0.154 | 0.092 | 0.265 | 0.280 | 0.084 | 0.000 |  |  |  |  |  |  |  |  |  |  | **Ko** |
| 0.138 | 0.094 | 0.233 | 0.227 | 0.064 | 0.054 | 0.000 |  |  |  |  |  |  |  |  |  | **Ka** |
| 0.137 | 0.139 | 0.263 | 0.269 | 0.020 | 0.068 | 0.049 | 0.000 |  |  |  |  |  |  |  |  | **La** |
| 0.182 | 0.022 | 0.047 | 0.056 | 0.152 | 0.082 | 0.098 | 0.108 | 0.000 |  |  |  |  |  |  |  | **Mt** |
| 0.153 | 0.133 | 0.272 | 0.262 | 0.084 | 0.093 | 0.012 | 0.060 | 0.130 | 0.000 |  |  |  |  |  |  | **Ya** |
| 0.123 | 0.133 | 0.262 | 0.275 | 0.021 | 0.064 | 0.038 | 0.006 | 0.114 | 0.049 | 0.000 |  |  |  |  |  | **Zi** |
| 0.167 | 0.040 | 0.131 | 0.136 | 0.117 | 0.037 | 0.076 | 0.088 | 0.023 | 0.124 | 0.088 | 0.000 |  |  |  |  | **Fi** |
| 1.098 | 1.480 | 1.403 | 1.458 | 1.500 | 1.597 | 1.342 | 1.399 | 1.409 | 1.374 | 1.427 | 1.492 | 0.000 |  |  |  | **Gg** |
| 1.688 | 1.803 | 1.648 | 1.748 | 1.981 | 1.928 | 1.329 | 1.886 | 1.749 | 1.886 | 1.915 | 1.851 | 1.322 | 0.000 |  |  | **Ta** |
| 0.056 | 0.271 | 0.321 | 0.371 | 0.157 | 0.209 | 0.192 | 0.179 | 0.229 | 0.209 | 0.170 | 0.217 | 1.196 | 1.683 | 0.000 |  | **Ch** |
| 0.828 | 1.246 | 1.150 | 1.250 | 1.252 | 1.275 | 1.174 | 1.242 | 1.162 | 1.205 | 1.220 | 1.226 | 0.412 | 1.220 | 0.912 | 0.000 | **Tu** |
