# Supplementary figures and images for: Genetic diversity of Nile tilapia (Oreochromis niloticus) populations in Ethiopia: insights from nuclear DNA microsatellites and implications for conservation
Source: BMC Ecol Evol. 2021 Jun 7;21:113. doi: 10.1186/s12862-021-01829-2 (PMC8183085; doi:10.1186/s12862-021-01829-2)

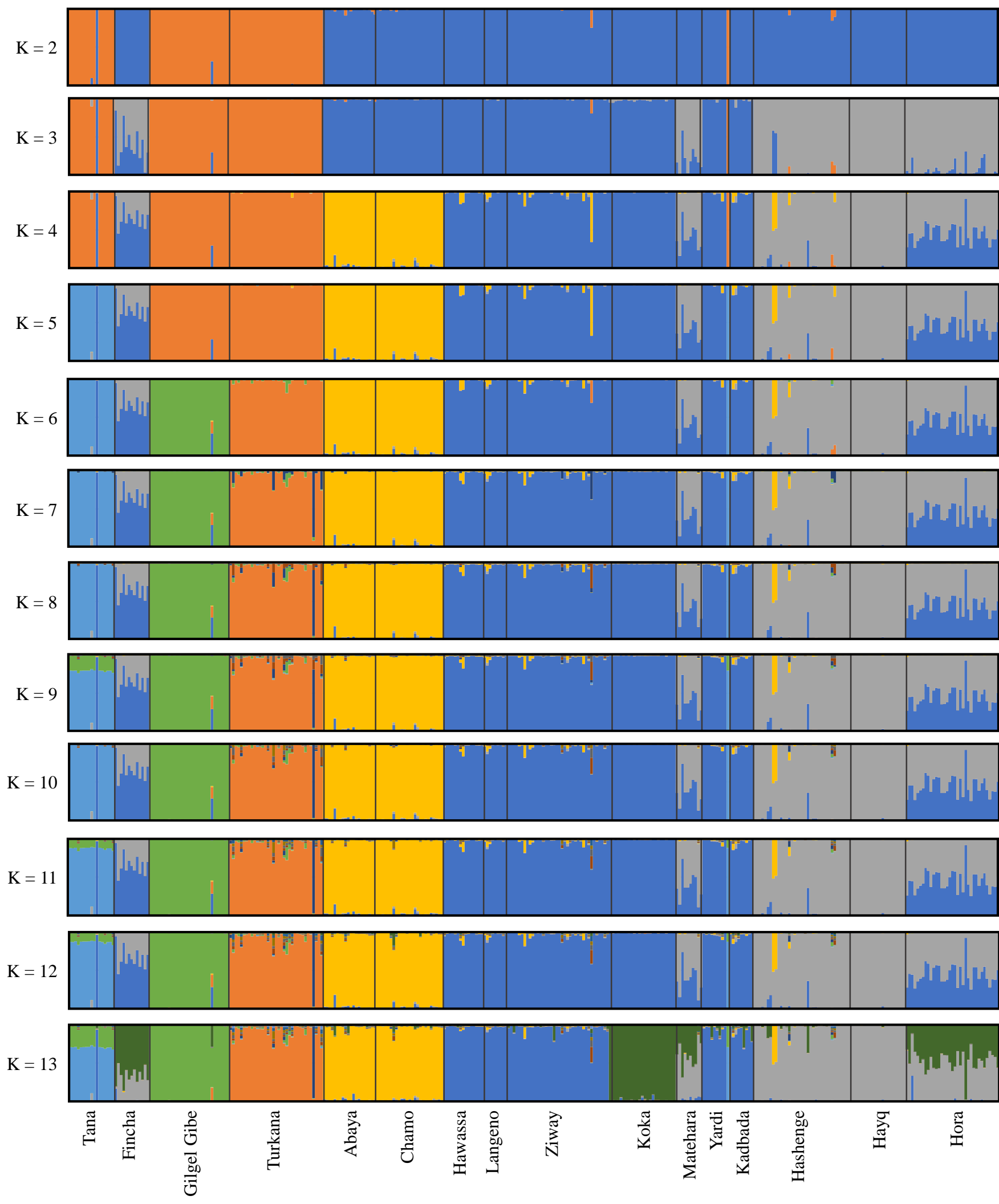

Supplement: Supplementary file 2 — Additional file 2: Figure S1. Structure analysis (admixture model) for all Nile tilapia samples investigated for all K values up to 13. [file 12862_2021_1829_MOESM2_ESM.pdf]

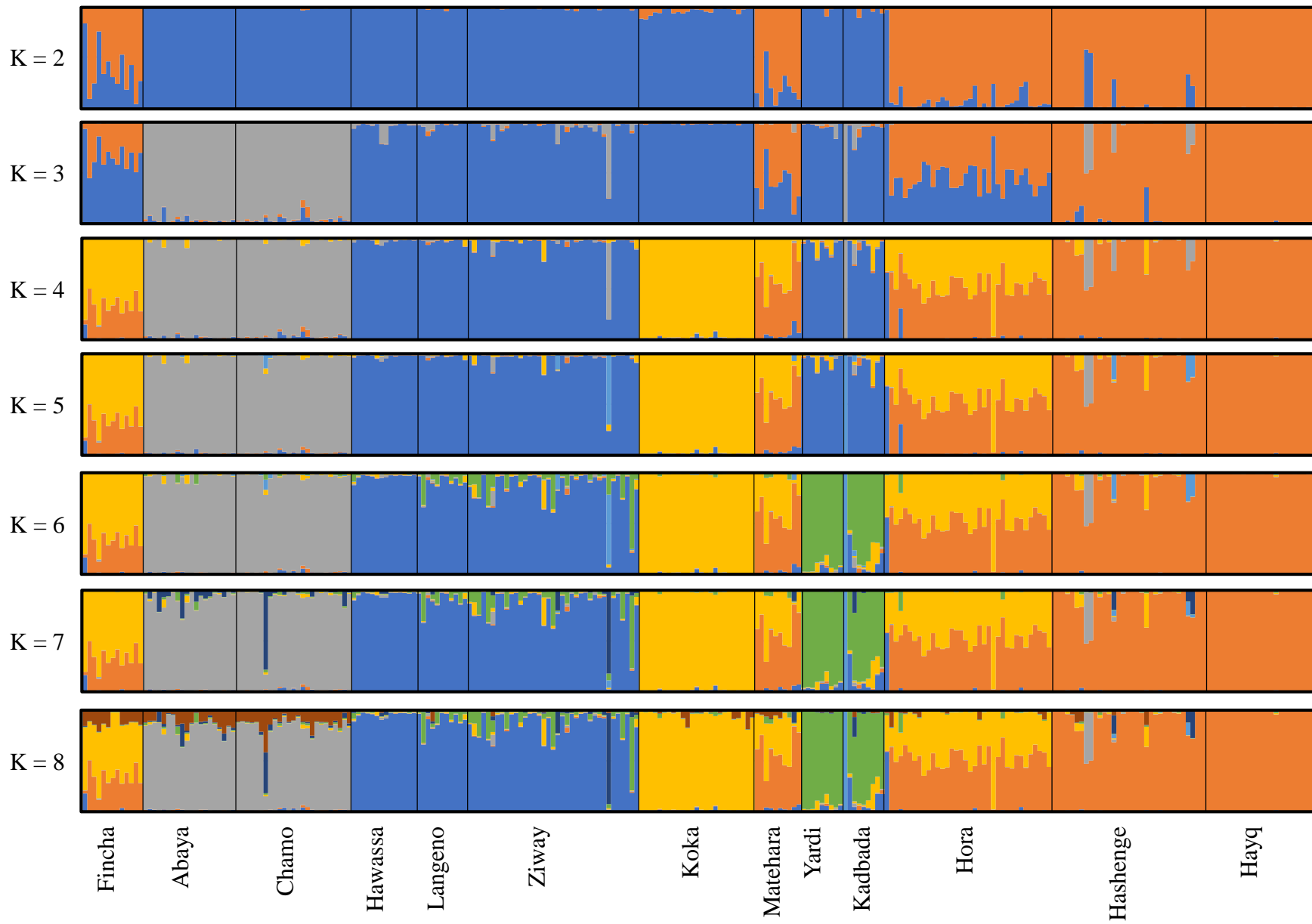

Supplement: Supplementary file 3 — Additional file 3: Figure S2. Structure analysis (admixture model) excluding the most divergent populations from Omo-Turkana (Gilgel Gibe, Turkana) and Lake Tana for all K values up to 8. [file 12862_2021_1829_MOESM3_ESM.pdf]
