# Supplementary material for: Genetic diversity of Nile tilapia (Oreochromis niloticus) populations in Ethiopia: insights from nuclear DNA microsatellites and implications for conservation
Source: BMC Ecol Evol. 2021 Jun 7;21:113. doi: 10.1186/s12862-021-01829-2 (PMC8183085; doi:10.1186/s12862-021-01829-2)
Supplement: Supplementary file 4 — Additional file 4: Table S2. Characteristics of microsatellite loci used to genotype Nile tilapia populations in Ethiopia. [file 12862_2021_1829_MOESM4_ESM.docx]

Additional materials

Table S2. Characteristics of microsatellite loci used to genotype Nile tilapia populations in Ethiopia.

| Locus | F: Forward Primer sequence (5’-3’) | R.motif |  |  | Locus | F: Forward Primer sequence (5’-3’) | R.motif |
| --- | --- | --- | --- | --- | --- | --- | --- |
|  | R: Rivers primer |  |  |  |  | R: Rivers primer |  |
| Ti_1 | F: TTA TCACTGCTGAACGTCTT | (TGG)7 |  |  | Ti-17 | F:AACTGAAGA AGAAGC CTTGG | (GGAA)47 |
|  | R:GTTTTGGCTGCTACACATTC |  |  |  |  | R:ATCATCTTCCTCTACTGCCT |  |
| Ti_6 | F: CAGCTCTCATGAACACTTGA | (AAC)21 |  |  | Ti-41 | F:TCGCAGCTGCTCCTGTTTAA | (AAAC)26 |
|  | R:ACCCATAAATCACACCAGTC |  |  |  |  | R:TTGTGCACGTGGACATGTTG |  |
| Ti_55 | F: GAGCCCAGACAGCAGACAAT | (TCTA)31 |  |  | Ti-13 | F:AATCCGTTAGCTGCAGATAG | (ATGG)8 |
|  | R:AGGACCTTCTATGGCCCTGT |  |  |  |  | R:GCTGATTAA ACACAAAGTTGG |  |
| Ti-49 | F: TCGAAGTAGCGTGGAAAACCT | (TGGT)9 |  |  | Ti-54 | F:TTTCTTGCCAGCAAAAACAGT | (GGGGAT)30 |
|  | R:ACAACAACAACAGGTCGGGA |  |  |  |  | R:CAGATTCTTCCAGTGCTTGTGC |  |
| Ti-27 | F: CTG TCT TTC TTG ATG TGG GA | (TTTGG)7 |  |  | Ti-50 | F:CCTGTGACAGACTGGTGACC | (ATGGGG)10 |
|  | R:ATG CAC AAA TTT TAA GGG CC |  |  |  |  | R:ACACTGATGCGGTTTACGGT |  |
| Ti-34 | F: GCT TAC AGT ACA TTG TGT GC | (TCTCT)41 |  |  | Ti-5 | F:AAGGAGGATGATCAGGACAC | (CA)10 |
|  | R:CTG ATG AGA AAA ACA GAC GC |  |  |  |  | R:AGACCTCCACTGTGATCTTA |  |
| Ti-15 | F: GCT GTG ATC ATC TGG AGA AA | (TGGC)10 |  |  | Ti-24 | F:ACTGACAACATAAAGACATATGA | (TTAC)28 |
|  | R:AGG ATC TAG AAC CTC CAA CC |  |  |  |  | R:CACAGTTTFAATCCACCATC |  |
| Ti-8 | F: CTG AAG TCC TGC TGA GAT TT | (AC)19 |  |  | Ti-28 | F:TGT CTT GGG ATT TGA GAT CA | (ATTCA)11 |
|  | R:CAT TGT TCT TGG CAC CTC TA |  |  |  |  | R:CGG AGG TTT CTT CCT GTT AA |  |
| Ti-32 | F: CAG GAA ATG GCT CCA AAA TG | (AAAAT)46 |  |  | Ti-9 | F:CTC AGT GAC GAA GCC AAA | (AC)8 |
|  | R:TTG TAG CTA GGA ATC AGT GC |  |  |  |  | R:CCT R:GGC AAT CAA AAG AACAA |  |
| Ti-39 | F: TACCTGCCAGTCATGTGCTG | (ATGGGG)17 |  |  | Ti-4 | F:TGT GCA GAA TAG AAT AGC CC | (GGT)26 |
|  | R:TGCTCAGACTGGTCCCTTCT |  |  |  |  | R:GAA AGG AAA AAT GTT GGTGGT |  |
| Ti-61 | F:G CTACACAGGAAAGCAGAGC | (TGGGGA)13 |  |  | Ti-2 | F:TTCTGGGCTAACACACAAG | (AC)28 |
|  | R:ACTCAATGCTGGACGTGACC |  |  |  |  | R:AAGGTGTCACACAGTTTAGG |  |
| Ti-51 | F: TGCTAAACGCCAGCTGATGA | (TGGT)9 |  |  | Ti-26 | F:ATT GCT TCA TCC CTT GAG TT | (ACAA)13 |
|  | R:TTACCACACGATGTCGCAGG |  |  |  |  | R:ACACGG AAA ACC TAA TGA CA |  |
| Ti_59 | F:ATGGACTTAAGCTGCACCCC | (AGGGA)18 |  |  | Ti-35 | F:TCA ACC ACA AAC TCC TCT TT | (AAAAGG)34 |
|  | R:TGAGCATTTGACCCCAGCAT |  |  |  |  | R:AAA CTA AGT GCA GCT CAT GA |  |
| Ti-43 | F:ATTGCCATCACCAGGAACCA | (GGAATA)37 |  |  | Ti-12 | F:GCCACCAAAATATTCGTGTT | (TAC)31 |
|  | R:TGCTAGCCCAGAGCATTTGA |  |  |  |  | R:CCATGTTCTGTCTCCTTGAA |  |
| Ti-57 | F:CAGTGGGAGGAAGCTCCAAA | (TCCA)12 |  |  | Ti-52 | F:GAGAAACGTCCAGTGGCAGA | (TAT)9 |
|  | R:GCTGCATGGATCCAATAGGC |  |  |  |  | R:TTTCGATCTGCTGCCCCTTT |  |
| Ti-6 | F:CAG CTC TCA TGA ACA CTT GA | (GGA)26 |  |  | Ti-33 | F:GCT TAT GGC TGT ATG GAG TT | (TTCAA)12 |
|  | R:ACC CAT AAA TCA CAC CAG TC |  |  |  |  | R:CGA CTT CTG TTG TGA TTT GG |  |
| Ti-18 | F:AGCAAGTGAGATAAGCACTG | (ATCT)38 |  |  | Ti-7 | F:TCT TTG TGT CAG AACTGTGT | (AC)18 |
|  | R:TACATAGCAGTGCAGTTTGC |  |  |  |  | R:ACTCTG CTTTTAGCCAATCA |  |
| Ti-60 | F:GAGCCGCCATAGTGTCACTT | (ATCC)16 |  |  | Ti-31 | F:GAA ACT ATCCAC AGAAGCCA | (CTAAT)23 |
|  | R:CCTGCTCTCACTCAAAGAGGG |  |  |  |  | R:AGG CTTCTTACAGTTGGATG |  |
| Ti-56 | F:TGCAGTGAATTTGGCACCTG | (TGGTT)13 |  |  |  |  |  |
|  | R:AGCCTGAGATACCTGTGCCT |  |  |  |  |  |  |
